# Microdialysis and microperfusion electrodes in neurologic disease monitoring

**DOI:** 10.1186/s12987-021-00292-x

**Published:** 2021-12-01

**Authors:** Luke A. Stangler, Abbas Kouzani, Kevin E. Bennet, Ludovic Dumee, Michael Berk, Gregory A. Worrell, Steven Steele, Terence C. Burns, Charles L. Howe

**Affiliations:** 1 School of Engineering, Deakin University, 3216 Geelong, Victoria Australia; 2 Division of Engineering, Mayo Clinic, 55905 Rochester, MN USA; 3 School of Medicine, Deakin University, 3216 Geelong, Victoria Australia; 4 Department of Neurology, Mayo Clinic, 55905 Rochester, MN USA; 5 Department of Neurosurgery, Mayo Clinic, 55905 Rochester, MN USA; 6 Division of Experimental Neurology, Mayo Clinic, 55905 Rochester, MN USA; 7 Center for MS and Autoimmune Neurology, Mayo Clinic, 55905 Rochester, MN USA

## Abstract

Contemporary biomarker collection techniques in blood and cerebrospinal fluid have to date offered only modest clinical insights into neurologic diseases such as epilepsy and glioma. Conversely, the collection of human electroencephalography (EEG) data has long been the standard of care in these patients, enabling individualized insights for therapy and revealing fundamental principles of human neurophysiology. Increasing interest exists in simultaneously measuring neurochemical biomarkers and electrophysiological data to enhance our understanding of human disease mechanisms. This review compares microdialysis, microperfusion, and implanted EEG probe architectures and performance parameters. Invasive consequences of probe implantation are also investigated along with the functional impact of biofouling. Finally, previously developed microdialysis electrodes and microperfusion electrodes are reviewed in preclinical and clinical settings. Critically, current and precedent microdialysis and microperfusion probes lack the ability to collect neurochemical data that is spatially and temporally coincident with EEG data derived from depth electrodes. This ultimately limits diagnostic and therapeutic progress in epilepsy and glioma research. However, this gap also provides a unique opportunity to create a dual-sensing technology that will provide unprecedented insights into the pathogenic mechanisms of human neurologic disease.

## Introduction

The United States Center for Disease Control (CDC) estimated in 2015 that 1.2% of the American population has active epilepsy, including approximately 3 million adults and half-a-million children [1]. Conservatively estimating that 20% of such patients have medically refractory epilepsy, this leaves roughly 700,000 patients in need of neurosurgical procedures or other novel strategies to achieve disease control [2]. Notably, CNS neoplasms comprise the underlying seizure focus in a subset of patients with intractable epilepsy. While some CNS neoplasms are benign, indolent, or surgically curable, most primary CNS malignancies remain incurable. Indeed, the most common adult primary brain tumor is glioblastoma (GBM), which is typically fatal in just over a year from diagnosis [3], with under 5% of patients surviving 5 years [4]. The development of new treatments for glioma has remained disappointing, with no new drug shown to improve survival since temozolomide was introduced in 2005 [5]. Therefore, at present, the therapeutic toolbox for both epilepsy and glioma are inadequate.

Paramount in the immediate surgical management of both epilepsy and glioma is optimally balancing the preservation of neurocognitive function while simultaneously achieving maximal safe resection of epileptogenic or tumor-infiltrated brain tissue. However, some gliomas and epileptic foci cannot be effectively removed without unacceptable loss of neurocognitive function. In such cases, neurosurgical avenues may still be pursued for diagnostic or therapeutic procedures, spanning biopsy, electrocorticography from subdural or penetrating electrodes (ECoG), stereotactically placed brain-penetrating electrodes (sEEG), and, less frequently, focal CNS delivery of therapies via convection-enhanced perfusion or chronic implantation of stimulation-based devices to mitigate propagation of seizures.

Human neurological diseases are notoriously challenging to accurately model in vitro or in preclinical models. However, collection, identification, and quantitation of extracellular biomarkers from the diseased human brain may offer the potential to enhance our understanding of pathogenic mechanisms and thereby accelerate the development of much-needed therapies [6]. Neuronal activity has recently been shown to facilitate glioma progression [7], but at the same time certain electrical stimulation paradigms attenuate tumor growth by increasing chemotherapeutic efficacy [8]. Moreover, seizure activity may both result from and lead to neurochemical and metabolic aberrations in the brain [6]. As such, the coordinated collection of electrical and biochemical information in these diseases may expose novel cause-and-effect relationships. The capture of correlated or interdependent neurochemical and electrical fluctuations may yield hypotheses relevant to the treatment of both epilepsy and glioma. Moreover, since electrodes can both record and stimulate, and since microdialysis or microperfusion can both sample and focally introduce agents into the extracellular biochemical environment, the combination of these modalities robustly expands the breadth of therapeutic hypotheses that can be directly tested in a patient-specific manner explicitly within diseased human CNS tissue.

Since biochemistry and electrical activity are the currencies of neurophysiology, applications of purpose-built technologies could extend beyond epilepsy to include patients undergoing neurosurgical procedures for stroke, traumatic brain injury, Parkinson disease, and other neurological and neurodegenerative conditions. Since effective technologies for concurrent biochemical and electrical interaction within the CNS are a prerequisite to progress in this arena, we address the key engineering-related factors of relevance to developing optimal dual-purpose devices, first addressing microdialysis and microperfusion separately before reviewing precedent efforts to combine these with invasive EEG recording electrodes.

## Comparisons between microdialysis and microperfusion

**Fig. 1:**
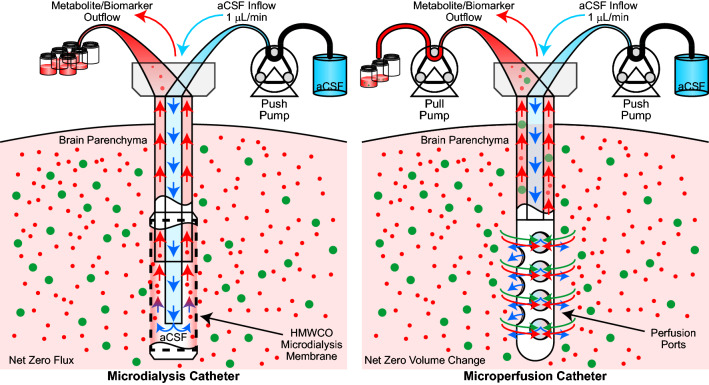
Microdialysis and microperfusion systems. The major features of microdialysis and microperfusion devices include cylindrical probes, reservoirs for metabolites and biomarkers, pumps to drive perfusion fluid flow and control fluid fluctuation into the tissue. Exclusive to microdialysis is a membrane that separates and protects the peri-probe tissue from the flowing perfusate. Microperfusion incorporates a secondary pull pump to draw perfusate from the catheter and perfusion ports, allowing larger molecules and compounds to diffuse into the collection fluid

Microdialysis and microperfusion are similar biomarker collection techniques that comprise a porous partition between the surrounding tissue microenvironment and perfusate flowing within the sampling device, allowing extracellular parenchymal compounds to diffuse down a concentration gradient across the partition [9] (Fig. 1). Many system designs include at least one precision pump responsible for propelling biomarker-free perfusate or microdoses of pharmaceutical candidates [10] from a reservoir to the diffusion probe where dialysis occurs [11]. The biomarker-rich perfusate, consequently termed dialysate, then continues to a microvial for stabilization and storage, prior to identification and quantitation of compounds such as pharmacological molecules, neurotransmitters, antibacterials, cytotoxic agents, cytokines, and metabolites [9]. Small molecules, peptides, and proteins are characterized in the dialysate using biochemical analysis, enzyme-linked immunosorbent assay, or liquid chromatography and mass spectrometry [12, 13].

A primary strength of these technologies is the ability to continuously collect perfusate using minimally invasive implantation [9] of probes that permit the analysis of biomarkers over the course of events such as electrical stimulation or during systemic or localized administration of pharmacological candidates [14–17]. Such continuous collection is also regularly used to monitor the effects of the implanted probe on the biochemistry of the peri-probe tissue [18–21]. An additional strength of microdialysis and microperfusion is the potential to streamline the determination of effective drug dosing, especially for compounds such as antibiotics, through localized dosing at micro levels [12]. Microdialysis and microperfusion provide a platform for baselining an analyte within a targeted area while inducing no net biological fluid or tissue loss, a significant advantage over the use of surgical resection for tissue sampling [22]. However, both sampling approaches are plagued by skewing of biomarker collection induced by tissue damage during implantation [20, 23–26], and both approaches require complicated calibration techniques to accurately determine recovery rate, defined as the percentage of collected biomarker relative to the true tissue concentration [27].

### Microdialysis and microperfusion in contrast

**Fig. 2:**
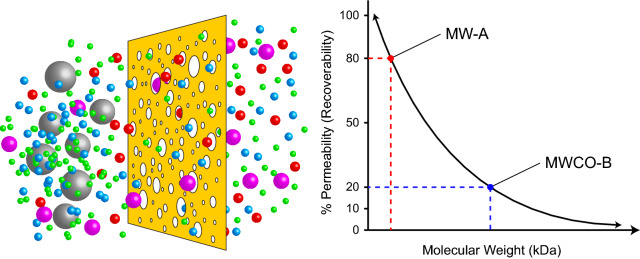
Microdialysis membrane molecular weight cut-off. Recovery rate across a defined MWCO membrane can be approximated by an exponential function that decreases as the molecular weight of the sampled compound increases [137, 138]. Mechanistically, this is related to the presence of progressively fewer large-diameter pores in the membrane relative to small-diameter pores, resulting in reduced probability of large molecule permeation. The largest pore size sets the absolute cut-off for permeability, but the effective cut-off is considerably smaller. The manufacturer specified MWCO for a membrane is a single point along a spectrum where compounds of a known molecular weight experience a specific transmission rate across the membrane, typically 10-20% (or conversely, 80-90% retention or permeability resistance). Therefore, to achieve high recovery (>80%) of a desired molecule with a specific molecular weight (MW-A), a membrane with a much larger MWCO must be employed (MWCO-B)

Microdialysis techniques progressed substantially in the 1990s and have been validated repeatedly thereafter, although with decreasing frequency in the last 10–15 years, providing foundational data and reliability as a developed science [9, 11, 14–16, 28]. The most significant distinctive quality of microdialysis in contrast to microperfusion is the incorporation of a semipermeable membrane to control the selection of sought after biomarkers based upon molecular weight cutoff (MWCO), measured in Daltons [21]. At any designated MWCO, roughly 80–90% of the molecules at that particular molecular mass are unable to transverse through the membrane while molecules of lower mass are able to pass through the membrane in a graded manner [9] (Fig. 2). The exact percentage of exclusion versus permeability for any specific molecular weight, however, varies between membrane manufacturers and does not behave like a step function. Also, the MWCO specified by the manufacturer may not provide an accurate prediction of the experimental recovery rate, even if the targeted compound has a molecular weight precisely at the MWCO. Selecting a membrane with a MWCO that is considerably larger than the molecular weight of the compound of interest is recommended to ensure measurable quantities of the compound are sampled experimentally [29]. However, while high molecular weight molecules such as cytokines, growth factors, and large neuropeptides have been successfully captured using high MWCO membranes [30], the recovery rates remain unpredictable. For example, the estimated recovery of cytokines across a 100 kDa MWCO membrane is only 1–5% at normal flow rates, despite these factors having molecular weights that are generally under 25 kDa [31, 32]. A unique solution to this challenge is to change the effective gradient “drive” for the cytokine across the membrane by adding polymer microbeads coated with antibodies to the perfusate. This strategy has been shown to increase the relative recovery of some cytokines in vitro by upwards of 20-fold [31] and may be a viable method for increasing recovery of many proteins in vivo.

Other benefits of microdialysis include the relative absence of tissue damage or hydrodynamic disruption after implantation is achieved. In contrast to microperfusion, microdialysis membranes can isolate tissue structures from possibly detrimental hydrodynamic forces associated with perfusate flow and therefore can induce less overall tissue damage [10]. Finally, microdialysis setups typically require only one push pump to achieve adequate perfusion, due to the physical retention of perfusate within the probe preventing flow into the peri-probe tissue [33] (Fig. 1).

Conversely, microperfusion avoids the need for the dialysis membrane and maximizes biomarker collection via the use of open collection ports in the indwelling catheter. The indiscriminate pore structure of the microperfusion probe circumvents the reduced recovery rates associated with microdialysis membranes (Fig. 2) and overcomes failure to collect sought after biomarkers due to unpredicted molecular interactions between the analyte of interest and the membrane. These ports, typically less than 1 mm in diameter, allow compounds of any size to fluctuate into the perfusate [34] (Fig. 1). Microperfusion probes are more commonly developed in experimental labs and cleanrooms [35] than microdialysis probes due to the simplicity of creating fluid channels compared to complex microdialysis membranes. Several groups have developed unique probes in-house to evaluate the design efficacy of stereolithography and micromachining [36], droplet generators [35], and soft lithography [37] for probe construction. Others laser etch holes into existing catheters to simplify the fabrication process [38]. However, microperfusion probes require a secondary pull pump to ensure a zero net fluctuation of fluid from the probe into the interstitial space [33, 34, 39]. Open flow microperfusion (OFM) has been validated in several studies in human skeletal muscle [40], adipose tissue [40], and cerebral tissue (cOFM) [21], supporting the validity of the approach in vivo.

As described, both microdialysis and microperfusion have distinct advantages (Table 1). Microdialysis is a highly documented sampling technology that excels for the collection of molecules below a predetermined molecular weight using a one pump system [33]. Unfortunately, the difficulty in finding appropriate commercial membranes along with membrane biofouling often hinder experimental success [26]. Conversely, microperfusion employs a simpler probe construction to sample molecules of any size [33] but has less of a published presence, prolonging experimental procedures and slowing technical implementation since the technology is still developing. Additionally, microperfusion requires a pull pump as well as a push pump to adequately control fluid flow [33].

**Table 1:** Advantages and disadvantages of microdialysis and microperfusion

	Microdialysis	Microperfusion
Advantages	Strong literature foundation Able to target specific molecular weights Single pump configuration	Less well characterized Largely avoids biofouling Collects biomarkers of all sizes Simple probe construction Simple sterilization process
Disadvantages	Membrane biofouling Complicated probe construction Lack of commercial membrane sizes Membrane expense Membrane sterilization challenges	Tissue damage at high flow rates Secondary pull pump required

### Microdialysis and microperfusion probe design parameters

The most critical design parameter in the microdialysis system is the semipermeable membrane (Fig. 2), responsible for establishing diffusion of the targeted biomarker across a gradient and for maintaining a net-zero fluid volume fluctuation [27]. Similarly, the size and quantity of open ports that comprise the microperfusion diffusion area are the critical part of the microperfusion probe [36]. Critical microdialysis membrane characteristics include construction material, surface charges that inhibit compound adhesion and dampen the interaction of the membrane with the neuroinflammatory response, MWCO, and more intuitive parameters such as geometry, temperature, stability, and reusability [41].

A plethora of membrane materials have been developed and successfully implemented in preclinical and clinical studies, including poly(carbonate-ether), cuprophan, and poly(acrylonitrile) [42]. Other membrane material options include poly(carbonate), poly(arylethersulphone), poly(ethersulphone), poly(urethane), or cellulose [10, 43]. The membrane material properties can critically impact sampling efficiency for biomarkers such as peptides, due to charge interactions [44], or proteins, due to absorption on the membrane [45].

Apart from material, the total area where diffusion occurs as well as inlet/outlet positioning are also critical determinants of performance for both microdialysis and microperfusion. Larger diffusion areas improve recovery rate by naturally providing more surface area for concentration fluctuation, although the data is variable and dependent upon target compound [12]. Microfabricated probes can suffer from low recovery rates due to reduced surface area and minor variations in the fabrication process [36]. Inlet and outlet geometry can be configured using a concentric tube design or a side-by-side configuration according to fabrication limitations, and material resources, while the material, such as fluorinated ethylene propylene, polyimide, stainless steel, or fused silica can be tailored to considerations such as dielectric or stiffness requirements or susceptibility to MRI fields [20, 21].

### Microdialysis and microperfusion performance parameters

Flow rates in each technology are commonly in the 0.5–1 µL/min range [31, 46] but have been experimentally investigated down to 0.25 µL/h (~0.004 nL/min) [47]. The basic premise guiding flow rate decisions is that recovery rate or extraction coefficient is inversely related to volumetric flow rate [27, 31, 46].

Additionally, lower volumetric flow rates decrease the probability of tissue damage induced by impinging fluid forces as the perfusate passes the collection site [10]. Computational modeling indicates the potential for higher shear stresses in microdialysis as compared to microperfusion [48]. With regard to microperfusion, evidence suggests that ultralow flow rates induce minimal tissue damage [49]. There is evidence that ultralow microperfusion flow rates (less than 10 nL/min) induce localized neuroinflammatory responses, though reducing the flow rate even further, to less than 5 nL/min, was associated with a reduced response [47].

## Recovery rate calibration

The concentration of the targeted compound in the collected solution does not necessarily represent the concentration of that same compound in the tissue, because diffusion varies depending on the surrounding tissue properties and flow properties internal to the probe [9]. Despite the 80–90% nominal membrane resistance estimate at the defined membrane MWCO, calibration is necessary to accurately estimate actual tissue concentration using both microdialysis and microperfusion [40, 50]. It is important to recognize that the measured concentration of the collected biomarker is often much less than the “true” concentration in the brain because of the combined diffusion resistance of the dialysate, the membrane, and the tissue [27]. The relationship between the concentration in the dialysate, ${C}_{D}$, and the tissue, ${C}_{T}$, is mathematically characterized by the recovery rate, *RR*, and is dominated by the flow rate, $Q$, and resistances to diffusion, $\sum R$, as shown in the following equations [51]:

$$RR=\frac{{C}_{D}}{{C}_{T}}.$$

$$RR=1-exp\left[\frac{-1}{Q(\sum R)}\right].$$

Total resistance to diffusion, *R*, can be expressed as a series function:

$$\sum R={R}_{D}+{R}_{M}+{R}_{E},$$

where *R*_*D*_ is the resistance of the dialysate, *R*_*M*_ is the resistance of the membrane, and *R*_*E*_ is the resistance of the surrounding tissue or environment [51]. In vivo resistance is a dynamic and rapidly changing function of tissue metabolism, cellular and vasculature exchange, and true diffusion [51]. In vitro calibration values cannot be extrapolated to in vivo samples due to the behavior of the live tissue relative to a synthetic media [51]. Therefore, it is necessary to calibrate the recovery rate of microperfusion and microdialysis experiments to calculate the true concentration of the targeted compound in the surrounding media. The no-net-flux calibration approach, also termed the equilibrium method, along with the reverse dialysis method [9] and extrapolation to zero method [12, 52] are together the most common calibration techniques and are further explored below. However, these calibration methods are subject to adhesion of the target analyte(s) to the flexible cannula between the membrane and the collection reservoir and to other plastic surfaces involved in the closed system. This non-linear loss must be taken into consideration for accurate calibration and it is important that the field works toward a more thorough understanding of how analytes (ranging from small molecules to large protein complexes and vesicles) adhere to the device. The development of active anti-fouling and anti-adhesion probes is a critical need.

### In vivo no net flux calibration

**Fig. 3:**
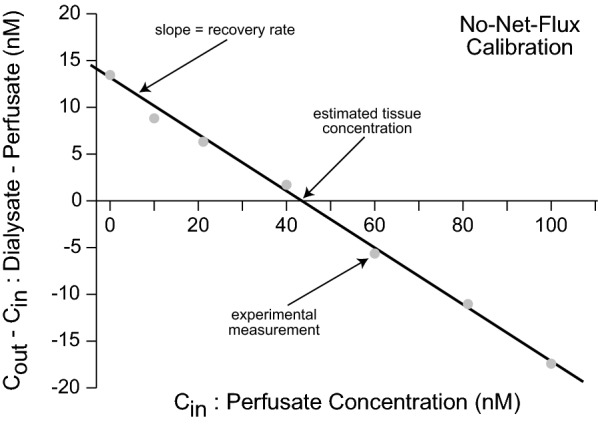
No-net-flux calibration method. Plotting the experimentally determined differences between output (C_out_ = dialysate) and input (C_in_ = perfusate) concentrations for a molecule of interest against the known input concentrations yields an estimate of actual tissue concentration at the point where the linear regression crosses the abscissa. In addition, the slope of the regression provides the recovery rate. While time consuming, this method provides an accurate estimate of tissue concentration with minimal a priori assumptions

The no-net-flux calibration method exploits repeated dialysate measurements while adding and incrementing the concentration of the targeted substance of choice into the dialysate inlet [40]. Measurement of the concentration is taken after the supplied dialysate is subjected to the unknown gradient between itself and the tissue [22]. After plotting the difference between input and outlet concentrations against the inlet concentration, the point at which the input concentration equals the outlet concentration (x-intercept) signifies the absolute concentration of the molecule in the peri-probe tissue, while the slope of the regression indicates the recovery rate [22, 40] (Fig. 3). A transient no-net-flux technique was also developed to decrease calibration duration for time-sensitive protocols and clinical trials [53].

### Retrodialysis calibration

The reverse dialysis method, termed retrodialysis, internal standard, or delivery method, has been well validated in vivo [22, 27, 46]. This procedure assumes that diffusion of any given molecule is equivalent in both directions across a membrane [22]. The perfusate is “spiked” with a known concentration of the analyte and the outlet (dialysate) concentration is measured and then used to back calculate the recovery rate using the following equation:

$$Recovery \,\, Rate \left(\%\right)= 100-\left(\frac{{C}_{Dial}}{{C}_{Perf}}*100\right).$$

A critical point for this calibration method is that diffusion is only equivalent in both directions if the initial concentration of the analyte in the peri-probe tissue prior to calibration is zero [22]. The presence of analyte in the peri-probe tissue prior to calibration will yield an underestimated recovery rate and an overestimated absolute concentration. This aspect makes the retrodialysis calibration method useful for measurement of exogenous drug levels but limited for measurement of endogenous biomarkers.

### Extrapolation to zero calibration

The extrapolation to zero method is executed by plotting the flow rate against the measured in vivo concentration over several different incremental flow rates [22]. The extrapolated intercept represents the concentration at a zero flow rate and therefore the absolute concentration of the substance of interest in the peri-probe tissue, with:

$${C}_{Dial}= {C}_{o}(1-{e}^{\frac{-rA}{F}}).$$

where *C*_*Dial*_ is the measured concentration of the analyte, *C*_*o*_ is the concentration of the substance around the probe, *r* is the mass transfer coefficient, *A* is the surface area of the membrane, and *F* is the experimentally defined flow rate [54]. Due to the nature of the exponential characterizing function, multiple very slow flow rates are recommended to minimize extrapolation error [22].

These calibration methods, although more popular in microdialysis, are also applicable to microperfusion, showing less than 10% variability between extrapolation to zero method and the no-net-flux method [34].

## Invasive consequences of implantation and biofouling

Published literature indicates several critical parameters to reduce tissue damage during probe insertion and post-implantation that will improve strategies for successful biomarker sampling. However, despite efforts to eliminate tissue damage, the nature of probe implantation is such that damage can only be mitigated, not prevented [18, 25]. For example, reduced analyte recovery [26] and acute changes in electrophysiological properties [16] as a consequence of probe implantation can be detected within minutes. In parallel, the neurochemical properties of potentially damaged tissue immediately adjacent to the probe differ from that of similar tissue as close as 1 mm away [25]. Implantation-induced contamination of the peri-probe tissue with blood-borne inflammatory factors is also a critical limitation for the accurate assessment of inflammatory cytokines and chemokines using either microdialysis or microperfusion [23, 55]. Mitigation of this issue may be realized by allowing the blood–brain barrier to heal prior to sampling, provided the experiment can be prolonged by up to two weeks [19, 21, 23]. Previous work has shown that the blood–brain barrier is a dynamic interface for the transportation of selective compounds [56], therefore emphasizing the need for the barrier to heal prior to sampling. If healing is not possible due to time constraints, then additional sampling may be necessary in order to isolate the influence of the barrier. For example, simultaneous testing in the blood, at the barrier, and in the brain, per se.

Factors that skew the collection of biomarkers due to tissue damage are the size [48, 57], geometry [58], and rigidity [59] of the implanted probe. The volume of damaged tissue increases with the cross-sectional size of the probe [39]. The cross-sectional geometry of the probe also plays a role in the variety and population of collected biomarkers [58–60]. Abnormally elongated cell nuclei are present directly adjacent to a cylindrical shaft but not to the thin edge of the same custom probe [58], and other probe geometries induce less neuronal loss along thin edges as compared to traditional cylindrical probes [58, 60]. Finally, a flexible polyimide electrode probe has been shown to limit the inflammatory response [59], suggesting that construction of flexible microdialysis and microperfusion probes may reduce tissue damage and improve accuracy and reliability.

Biosensor failures can also stem from biocompatibility failures such as biofouling [26]. The membrane biofouling layer that forms on biosensors tends to be avascular and fibrous, reducing analyte recovery gradually over time, in contrast to the acute effect mediated by tissue damage during probe implantation, which improves with time [26]. Implanted microdialysis probes have been shown to induce progressively increasing reactivity in both astrocytes and microglia in the probe vicinity over the course of a month, indicating biofouling rather than an invasive trauma response [20]. Biomimicry and microperfusion flow show strong potential for reducing biofouling due to the performance of biomimicry in the first 2 weeks post implantation [26] and 97% retained sensitivity of microperfusion flow technology in the first four hours after implantation [26].

Not only do the invasive consequences of probe implantation and biofouling affect the efficacy of biomarker collection, these factors may also impact underlying pathogenic mechanisms directly. Tissue sites surrounding microdialysis probes have shown elevated production of cytokines such as interleukin-1β (IL-1β) within one hour of implantation [61], IL6 within two days [62], and elevated astrocyte reactivity within one week [62]. These factors may suppress tumor growth or exacerbate peritumoral injury [63]. They may also promote neuronal function and synaptic maintenance [64–66] or elicit network dysfunction and seizures [67].

## Electrode design and performance

There are several design and performance parameters to consider in neural depth electrode development. Depth electrodes are categorically broken into microelectrode designs capable of capturing EEG data from single neurons [68, 69] and macro electrodes that collect signal over several square millimeters [70]. Microelectrodes are regularly fabricated through lithography techniques including sputtering or electron beam (e-beam), chemical vapor deposition, and etching [71–73] to capitalize on electrode density over small surface areas, although those processes can be extrapolated for macroelectrodes, as well [74]. For both electrode types, conductor materials tend to be platinum iridium [74], especially in patient studies [75–77], or titanium [78, 79], tungsten [80, 81], or biocompatible magnesium [72] surrounded by insulative polymers such as polyimide [73, 82, 83] and PDMS [72, 75].

Microelectrodes have benefits in precision data collection and the reduction of tissue response to implantation, just as miniature microdialysis and microperfusion probes reduce inflammatory responses that correlate with probe size [84]. Tissue disruption is in proportion to the cross sectional area of the probe [81]. After implantation, microelectrodes can be implanted to target specific cell layers [76] or provide localized data from sub-millimeter scale neuronal assemblies [77]. Multiple microelectrodes can monitor spatiotemporal patterns unlike a single macroelectrode [76] and can increase spatial density due to the nature of their size. Microelectrode implantation techniques may increase surgical risk to the patient while decreasing the accuracy of implantation [76, 85]. Lesion volume does not appear different between microelectrodes and macroelectrodes [85].

Conversely, macroelectrodes can provide similar data in terms of cellular spiking by delineating areas of cellularity [76, 86]. Macroelectrodes provide larger activation areas that linearly decrease radially away from the electrode in terms of stimulation [76]. Microelectrodes stimulate more cells relative to their occupied volume with activation profiles that decrease more rapidly away from the probe [76]. Macroelectrodes are preferred for long term implantation due to improved durability [86].

In terms of electrode performance, corrosion of electrodes and concomitant surface area reduction increases current densities and chemical reactivity and therefore skews data over time [87]. Regardless of electrode type, work has identified factors to improve biocompatibility and reduce corrosion and therefore collect more consistent data. Boron-doped diamond electrodes have been developed to improve electrochemical properties and improve corrosion resistance over several months [79, 88–91]. Platinum electrodes are recommended over stainless steel electrodes to maintain biocompatibility and minimize risk of signal drift [87]. Plating tungsten microelectrodes with platinum black coatings reduces impedance and improves post-stimulation cell activation [92].

Modifications of the insulation surrounding the electrodes can also improve electrode functionality and biocompatibility. Specifically, lowering polyimide curing temperatures leads to more stable chronic data collection [83]. Surface adhesion of biomolecules and polyurethane hydrogels promote biocompatibility by reducing protein absorption [93] and astrocyte attachment [94]. Lastly, reducing micromotion of implanted probes via probe geometry and flexibility decreases the magnitude of chronic gliosis [73].

## Previously fabricated microdialysis and microperfusion electrode systems

Several microdialysis and microperfusion electrodes have been designed and tested to tackle the *in vivo* sensing challenge of simultaneously measuring changes in biomarkers during passive electrophysiological recording or active electrical stimulation of brain tissue [16, 95–105]. Some of the more elementary designs involve two separate probes for neurochemical sampling and EEG recording [95, 96, 102, 104, 106–114]. Other designs successfully incorporate both technologies into a single probe [98–101, 103] or even fix separate probes together through various means [115–121]. The majority of these studies were conducted *in vitro* or in non-human animal models, with minimal evidence obtained from clinical studies to date [122, 123].

### Preclinical examples of dual-sensing systems employing microdialysis and microperfusion electrodes

The default setup for concurrently measuring neurochemicals along with EEG is commercially purchased microdialysis probes located separately from implanted stimulating/recording electrodes [96, 102, 104, 106–114]. Separate microdialysis and electrochemistry probes have been deliberately placed in rat striatal locations at least 2 mm apart to determine local gradients of dopamine and dopamine metabolites following amphetamine administration [102]. Most preclinical efforts measure dopamine or glutamate in rat brains where the probe separation distances are unspecified [106–108, 110–112]. Multi-electrode arrays have been used to measure local field potentials while simultaneously collecting extracellular analytes via microdialysis in opposing mouse brain hemispheres [113]. Another experiment measured EEG at the cortical surface [114] while simultaneously collecting small molecule biomarkers and metabolites by microdialysis in other locations. Lastly, one group fabricated and effectively used a microperfusion probe to measure glutamate fluctuations induced by a stimulating electrode in the opposing hemisphere of an anesthetized mouse [35].

To minimize the spatial gaps between the sEEG probe and the neurochemical sampling probe, several studies have employed a commercially available microdialysis cannula epoxied to an empty guide cannula to permit simultaneous collection of small molecules in proximity to a concentric bipolar electrode in the rodent caudate and nucleus accumbens targeting GABA and other amino acids [115, 119–121]. Others have attached recording electrodes directly to the microdialysis cannula to collect zinc ion [116, 117] or phenytoin for monitoring seizure activity [118]. A critical early study utilizing this methodology employed a flow-through hollow fiber with 50 kDa MWCO epoxied to a bipolar electrode to measure changes in taurine, glutamate, and glycine during seizures induced by intrahippocampal infusion of quinolinate [124]. To further minimize positional differences between the microdialysis probe and the electrodes, some setups incorporate conventional microdialysis with a concentric electrochemical electrode to measure neurotransmitters in two different modalities [125, 126].

### Clinical examples of dual-sensing systems employing microdialysis and microperfusion electrodes

Clinically, cerebral microdialysis has been performed with a hybrid invasive EEG electrode in neurosurgical patients with medically intractable epilepsy to delineate resectable seizure foci. In a seminal study from During and Spencer [122], a microdialysis probe was attached to a polyurethane/silastic brain-penetrating electrode (referred to as a depth electrode or stereo-EEG (sEEG) electrode) which was inserted into the hippocampus of patients with refractory epilepsy. In conjunction with subdural strip electrodes, this study captured changes in glutamate and GABA after electrophysiologically defined seizures. In another critical study [123], flexible polyurethane invasive, tissue-penetrating electrodes (Ad-Tech) with 6 or 7 platinum macroelectrodes spaced along the shaft and four to nine 40 μm diameter platinum/iridium microwires inserted within the lumen extending 4-5 mm beyond the probe tip were used for electrophysiological recording. A 200 μm diameter cuprophan microdialysis membrane (Akza Nobel Faser AG) was inserted through the same lumen and extended 10 mm beyond the tip of the electrode, revealing changes in multiple amino acids during seizures and cognitive tasks. While various other clinical strategies have been employed for dual electrophysiology and neurochemical sensing in humans, the intralumenal microdialysis-penetrating electrode configuration remains the state-of-the art [127].

### Relevant patents and commercial systems

A US patent from 1995 [98] claims a dialysis electrode device in which a semipermeable membrane forms the outer wall of a hollow probe while a working electrode, reference electrode, and counter electrode are fed through the top of the probe head, shielding the functional electrode from the tissue environment [98]. However, this device is not truly a dual sensing platform, as the perfusate is not under flow during measurement and the system uses an enzymatic electrode that can only assess one analyte at a time. Another US patent, from 1994 [128], claims a more generalized approach in which a cylindrical microdialysis probe ensheaths an internal “primary probe” that may collect electrophysiological data. Even more generally, a patent from 2003 claims a multi-lumen catheter system that can simultaneously support multi-modal interrogation of tissues, including the brain [129]. A search for patents incorporating “microdialysis”, “brain”, and “electrode” failed to identify any specific inventions that collect dialysate under flow either via microdialysis or microperfusion coupled with simultaneous collection of electrophysiological data in a form factor that is attentive to the size and geometry limitations discussed above.

A commercial version of the intra-lumen microdialysis-depth electrode system, employed by Spencer et al. [127], was manufactured by Ad-Tech with the explicit purpose of temporally synchronizing microdialysis, invasive EEG, and single neuron recordings. A standard Spencer depth electrode with 8 platinum macro-electrodes and 8 platinum micro-electrodes was modified such that 4 rows of perforations spaced 90 degrees circumferentially were placed between two sets of micro- and macro-electrodes at a location between approximately 4 mm and 20 mm from the electrode tip. The electrode was further modified at a point distal to the recording area (extracranially located after insertion of the electrode) such that 20 kDa or 100 kDa MWCO microdialysis catheters could be inserted (M Dialysis 70 and 71 brain catheters). This configuration allowed macroscopic perfusion through the perforations so that extracellular fluid was freely in contact with the microdialysis membrane within a region straddled by recording electrodes. Unfortunately, for unknown reasons, this device is no longer available from Ad-Tech.

## Discussion and open challenges

While microdialysis and microperfusion are closely related in construction, configuration, and purpose, the benefits of microperfusion generally outweigh the advantages of microdialysis. Microperfusion provides a simpler design allowing end-users to avoid limited commercial options for MWCO, thereby yielding versatility in probe geometry and perfusion port design through in-house fabrication that often requires minimal overhead equipment. Microperfusion casts a wider biomarker net and supports improved sensitivity to large compounds that have difficulty diffusing across the high-resistance dialysis membrane [31], but cannot restrict molecules of a particular molecular weight that might reduce signal-to-noise in the analyte analyses. Notably, microperfusion can collect biomarkers to reveal preclinical and clinical phenomena that would otherwise not pass through the semipermeable membrane of microdialysis. Such novel biomarkers include extracellular vesicles and other membrane-bounded structures as well as multi-molecular complexes and aggregates.

Calibration is required and should be conducted *in vivo* using the no-net-flux method if time permits, or via the extrapolation to zero method, but likely not the reverse dialysis method, assuming that the compound of interest exists in the peri-probe tissue. Despite the utility of these calibration techniques under well-controlled conditions, application of these methods within the dynamic clinical setting is challenging. This is due to insufficient time to vary parameters, limited time to achieve steady-state conditions, and concerns regarding introduction of exogenous materials into the host tissue. In practice, such conditions cannot be achieved in patients and current calibration methods are inadequate to improve estimates under non-steady state conditions. Aside from the relative fluctuations in collected biomarkers, application of microdialysis and microperfusion under real-world clinical conditions – especially within the context of the CNS – will require the development of novel techniques to provide accurate estimates of absolute tissue concentrations. Both microdialysis and microperfusion provide effective ways to measure biomarker fluctuations over the course of time of epileptic events but fall short in terms of measuring absolute concentrations in the clinical setting. Furthermore, the relationship between neurochemical fluctuations and invasive electrophysiology (local field potentials, multi-unit and single neuron activity) measurements is experimentally characterized by a lag due to the time that it takes compounds to diffuse through semipermeable brain tissue [51] as well as the time it takes electrophysiology signals to be transmitted and processed. Although the lag from collecting neurochemical samples can be several minutes [130], mathematical models exist for estimating molecular diffusion rates in brain tissue [131] and for correlating with electrophysiology signal processing [132, 133]. Additionally, data collected from microdialysis and microperfusion studies can be compared to the limited data of previous clinical studies as a means of validation [15]. Future microperfusion and microdialysis studies will benefit from additional published data sets correlating neurochemical biomarkers to brain electrophysiology in both healthy and diseased patients using traditional methods.

Inflammatory and reactive responses to the probe remain problematic despite the reduction of biofouling in the microperfusion realm [26]. While the inflammatory response skews acute data collection [16, 26] and biofouling reduces recovery over time [26], it is also evident that these responses affect the tumor microenvironment and induce seizure-like discharges. The presence of cytokines, especially factors such as IL-1β, near the probe implantation site have been shown to support immune reaction against the growth of tumor in other studies [63], suggesting a benefit of probe implantation in the vicinity of a tumor. Conversely, factors such as IL-1β and TNFα have the potential to directly drive aberrant discharges [134] and seizures [67] and may therefore exacerbate disease in patients with refractory epilepsy. Probe designs that emphasize minimally invasive techniques may mitigate some of the influence of inflammatory and tissue injury factors on the recovery of biomarkers. Regardless of how minimally invasive an implantation technique is, however, inflammatory tissue responses remain unavoidable without the development of active anti-inflammatory or anti-reactivity strategies. Successful mitigation steps also include implementation of miniaturized [57] flexible [59] or anchored [73] probes with flow rates beneath the threshold for perfusate pressure-induced tissue damage [135]. Ultimately, the widespread use of clinically-implanted catheters will be hampered until probe designs are developed that do not induce significant injury and inflammatory responses in the brain [59].

In terms of invasive electrophysiology electrodes, penetrating EEG electrodes are largely broken down into microelectrodes and macroelectrodes. Microelectrodes provide enhanced ability to monitor localized neuronal populations and single neuron activity compared to large-area macroelectrodes that sample the collective activity of large neuronal populations [77, 136]. Macroelectrodes may also provide more durability for long-term monitoring [86]. For both types, platinum-iridium electrodes show strong evidence of tissue compatibility [87, 122, 123] and cleanroom fabrication processes can promote further biocompatibility.

Several studies have been conducted to collect neurochemical biomarkers concurrently with invasive EEG data. Incorporating multiple probes, especially commercially purchased probes, can expedite experimental progress at the expense of collecting data that overlooks localized neurochemical fluctuations. Most of these published experiments have been conducted in rodents while utilizing commercially-available microdialysis probes in separate locations from the brain-penetrating electrodes [96, 102, 104, 106–114] despite evidence that extracellular biomarker diffusion is severely limited by tissue resistance [51, 110]. Therefore, it is crucial that the neurochemical sampling probe is spatially coincident with the target neurons to properly correlate fluctuations in analytes with electrophysiology data. Most studies performed in rodent models have not captured such physically coincident analyte and electrophysiological data. The setups that successfully collected neurochemical data and electrophysiology data from the same probe employed small-molecule microdialysis and targeted neurotransmitters such as GABA and glutamate and other amino acids [115, 119–121] with improved spatial resolution [115]. These preclinical setups did collect relatively coincident data correlating neurotransmitters to seizure activity but were limited by the lack of sensitivity to large compounds due to the nature of microdialysis.

Clinically, the studies mentioned above utilized microdialysis in conjunction with invasive electrophysiology electrodes to correlate seizure activity to GABA, glutamate, and other small molecules [122, 123]. The separation distances between the location of analyte sampling and EEG sampling were not well specified [122] and could be upwards of 10 millimeters depending upon implantation deflection [123]. As with the preclinical experiments, the focus was mostly limited to seizures and associated neurochemicals, with no evidence regarding larger molecules. Ultimately, the simultaneous capture of large molecules and electrical fluctuations may yield hypotheses relevant to the treatment of both epilepsy and gliomas. The combination of these modalities will robustly expand the breadth of therapeutic hypotheses that can be directly tested in a patient-specific manner explicitly within diseased human CNS tissue.

Despite the plethora of previously developed and published microdialysis electrodes and microperfusion electrodes, no known microperfusion electrode system has been developed for preclinical or clinical use. Thus, there is a significant unmet need for the development of novel microperfusion electrode dual-sensing probe designs that can concurrently collect large neurochemical biomarkers such as cytokines, metabolites, cell-free DNA, extracellular vesicles, and microRNA at the precise location of brain electrophysiology measurements (single neuron, multi-neuron, local field potentials, and EEG) monitoring. Coupled with high resolution electrophysiological monitoring spatially coincident with microperfusion ports, such a system will allow unprecedented insight into the dynamic environmental changes associated with neural function and dysfunction.

## Conclusion

Microdialysis and microperfusion are sampling techniques that may provide unique insights into neurologic disease microenvironments, especially when paired with invasive electrophysiology recordings. While both microdialysis and microperfusion have separate advantages, far more work has been done to validate preclinical and clinical use of microdialysis, leaving microperfusion as a relatively unexplored technology that may ultimately have more clinical potential and advantages. Calibration of both approaches is well documented and best conducted through the No-Net-Flux method, if conditions permit. Concerns regarding the tissue inflammatory response and biofouling may be addressed with new geometries and the development of active, rather than passive, anti-inflammatory properties built into the probe materials. Multiple previous endeavors have incorporated invasive EEG and neurochemical sampling into localized areas by fastening separate probes or designing one-off microfluidic electrodes to target dopamine and other neural biomarkers, mostly in small animal experiments and *in vitro* settings. The lack of adequate techniques for collecting neurochemical compounds such as cytokines and other large molecules, the absence of clinical *in vivo* validation testing of microperfusion electrodes, and the existence of large spatial separations between electrodes and perfusate collection locations results in a significant unmet gap between the available technology and the devices needed to support novel advances in epilepsy and GBM research.

## Data Availability

Not applicable.
